# Expression, regulation and targeting of receptor tyrosine kinases in esophageal squamous cell carcinoma

**DOI:** 10.1186/s12943-018-0790-4

**Published:** 2018-02-19

**Authors:** Manoj Kumar Kashyap, Omar Abdel-Rahman

**Affiliations:** 1 School of Life and Allied Health Sciences, Glocal University, Saharanpur, UP 247121 India; 2 Department of Applied Sciences and Biotechnology, Shoolini University of Biotechnology and Management Sciences, Solan, Himachal Pradesh India; 3 Clinical Oncology Department, Faculty of Medicine, Ain Shams University, Cairo, Egypt

**Keywords:** Esophageal adenocarcinoma, Esophageal squamous cell carcinoma, Tyrosine kinase receptor, Kinase activity, EGFR, VEGFR, AXL, PTK7, C-MET, ALK, And PDGFR

## Abstract

Esophageal cancer is one of the most common types of cancer, which is a leading cause of cancer-related death worldwide. Based on histological behavior, it is mainly of two types (i) Esophageal squamous cell carcinoma (ESCC), and (ii) esophageal adenocarcinoma (EAD or EAC). In astronomically immense majority of malignancies, receptor tyrosine kinases (RTKs) have been kenned to play a consequential role in cellular proliferation, migration, and metastasis of the cells. The post-translational modifications (PTMs) including phosphorylation of tyrosine (pY) residue of the tyrosine kinase (TK) domain have been exploited for treatment in different malignancies. Lung cancer where pY residues of EGFR have been exploited for treatment purpose in lung adenocarcinoma patients, but we do not have such kind of felicitously studied and catalogued data in ESCC patients. Thus, the goal of this review is to summarize the studies carried out on ESCC to explore the role of RTKs, tyrosine kinase inhibitors, and their pertinence and consequentiality for the treatment of ESCC patients.

## Background

Esophageal cancer (EC) is the 8^th^ most mundane malignancy and 6^th^ leading cause of death ecumenical. EC can be categorized into two subtypes predicated on histology: esophageal adenocarcinoma (EAC or EAD) and esophageal squamous cell carcinoma (ESCC) bearing different epidemiology and imperil factors [1]. ESCC and EAD are imposing together a major ecumenical health quandary. Both of these have peculiar geographic distribution as former is more prevalent in India, China, and Iran and later one is prevalent in North American, UK and Australia [2]. EAC arises from metaplastic Barrett’s esophagus (BE) and related to gastro-esophageal reflux (GER) and obesity. The most important risk factors for ESCC are alcohol and tobacco. Difference between the two subtypes is not limited to epidemiology or risk factors bur rather extends to treatment approaches [3].

The pathophysiology of different malignancies is driven in part by the growth factor receptors and growth factors mediated signaling. Among these signaling pathways, receptor tyrosine kinases (RTKs) are of special interest as these play an important role in the signaling of tumor cells, in different cellular processes like proliferation, migration, differentiation, cross-talk, metabolism and programmed cell death [4, 5].

RTKs are class of enzymes that lead to phosphorylation at the tyrosine (Y) residue of a protein using Adenosine triphosphate (ATP). The sequencing of the human genome led to identification of ~ 518 protein kinases [6]. Occurrence of TKs is restricted to metazoans only. Among 90 known TKs: 58 belong to RTKs and 32 to non-receptor tyrosine kinases (NRTKs). RTKs are activated by ligand binding to their extracellular domain. A number of proteomics studies have been carried out on ESCC [7–12], but only one study based on an in vivo labeling technique stable isotope labeling with amino acids in cell culture (SILAC) was focused on the phosho-tyrosine (pY) profiling using ESCC cell lines [13]. RTKs have been reported in a number of different malignancies including head and neck squamous cell carcinoma (HNSCC), oral squamous cell carcinoma (OSCC), lung adenocarcinoma, chronic myeloid leukemia (CML), and chronic lymphocytic leukemia (CLL).

### Different tyrosine kinases, their expression, regulation and targeting in ESCC

**Fig. 1:**
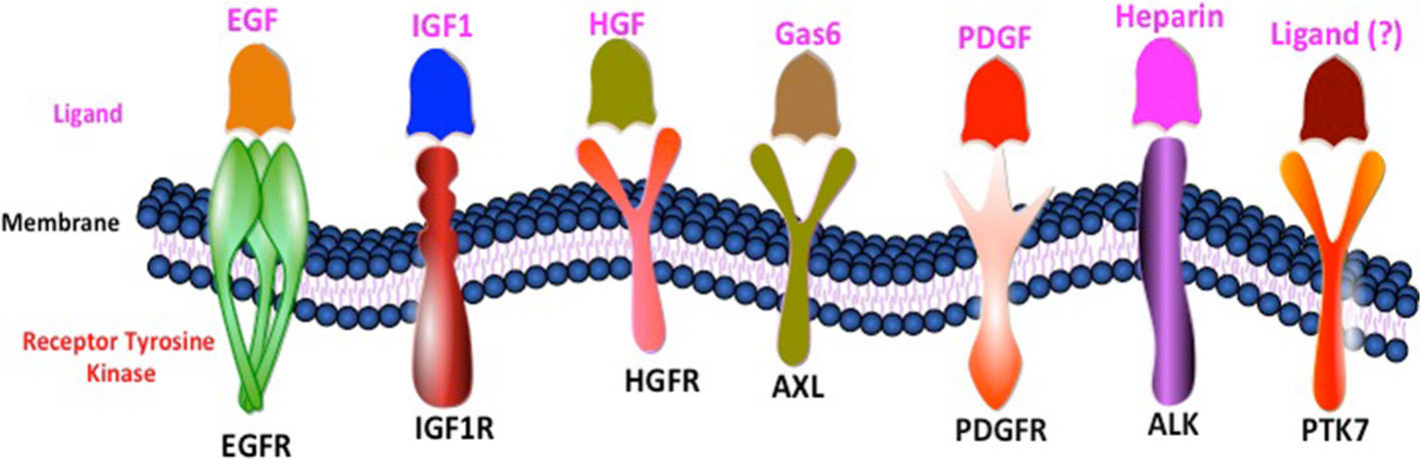
Different Receptor Tyrosine Kinases with their respective ligands reported in Esophageal Squamous Cell Carcinoma

A very first report for role of tyrosine phosphorylation was reported in 1989 by Ogawa et al. (1985), where they found an incrementation in levels of tyrosine phosphorylation in different cancers including EC utilizing monoclonal antibody against O-phosphotyrosine (PTYR) [14]. In recent years, a number of studies reported expression of different RTKs in ESCC (Fig. 1). The details about the architecture, domains, signal peptide and gene ontology based information has been provided for different tyrosine kinase receptors in Table 1. Additionally, a number of tyrosine kinases (TKs) have been assessed for their therapeutic value in ESCC either at in vitro or in vivo levels using tyrosine kinase inhibitors (TKIs). Here, we are presenting an update of the studies focusing on the expression of RTKs or studies where RTKs were targeted in ESCC (Table 2).

**Table 1:** Biological Characteristics of Some of the Receptor Tyrosine Kinases Reported in Esophageal Squamous Cell Carcinoma

Name of the receptor (HGNC Official Name)		Gene Symbol	Gene Locus	Ligand	Structural Feature of the Class		Primary Localization	Alternate Localization	Function
					Domain	Motif			
Epidermal growth factor receptor		EGFR	7p11.2	EGF	Three FU, one REC, one TM, and one TYK domain	One SP	Plasma Membrane	Endosome, Clathrin-coated vesicle, Cytoplasm	Stimulate proliferation of different cell types
Insulin like growth factor I receptor		IGF1R	15q26.3	IGF1/IGF2	One FU, one TM, one TYK, two REC, three FN3, and one TM domain	One SP	Plasma membrane		Regulation of cell growth and survival
MET proto-oncogene		MET	7q31	HGF	One TYK domain, One PSI, One SEMA, four IPT domains	One SP	Plasma Membrane	Cytoplasm	In biological processes like cellular proliferation, motility, migration, & invasion
Vascular endothelial growth factor receptor	FMS related tyrosine kinase 1	FLT1	13q12.3	VEGF	Five Ig_LIKE domains, two IGC2 domains, one TM domain, and one TYK domain	One SP	Plasma Membrane	Extracellular	Transmembrane receptor protein tyrosine kinase activity cell communication, signal transduction, and stimulate angiogenesis
	Kinase insert domain receptor	KDR	4q12	*VEGF*				Cytoplasmic	
Platelet derived growth factor receptor		PDGFRA	4q12	PDGF	Three Ig LIKE, one IGC2, one TM, and one TYK domain	One SP	Plasma membrane	Extracellular	Growth, differentiation and cell death controls
		PDGFRB	5q32						
ALK		ALK	2p23.2-p23.1	Heparin (an activating ligand)	One TM, one TYK domain, one LDLA, & one MAM	One SP, two NPXY	Plasma membrane	Cell surface	Embryonic brain development, Important role in the genesis and differentiation of the nervous system
Protein tyrosine kinase 7		PTK7	6p21.1	No known ligand (Context-dependent signaling switch for the Wnt pathways Wnt)	Five IGC2, two Ig LIKE, a TM, & a TYK domain	One SP	Plasma Membrane	Cytoplasmic expression	Cell adhesion, migration, polarity, proliferation, actin cytoskeletal reorganization and apoptosis

*FU* Furin-like repeats, *REC* cheY-homologous receiver domain, *TM* Tramsmembrane domain, *SP* Signal peptide motif, *NPXY* Asn-Pro-X-Tyr, *TYK* Tyrosine kinase domain, *LDLA* LDL receptors, the class A, *KRINGLE* Kringle Domain, *FZ* Frizzled domain

Note: primary and secondary localization of the receptor tyrosine kinases are either based on the information annotated in the Human Protein reference Database (HPRD, http: hprd.org) or the human protein atlas (HPA, https://www.proteinatlas.org)

**Table 2:** Summary of the selected clinical experiences with different agents targeting Receptor Tyrosine Kinases in ESCC

Study	Type of the study	Number of patients	Indication	Primary outcomes
i. EGFR-targeting agents:				
Dutton et al. (2014)	Phase III	Total number: 450 patients ESCC: 106	Gefitinib for esophageal cancer progressing after chemotherapy	The use of gefitinib as a second-line treatment in esophageal cancer in unselected patients does not improve overall survival (for all patients as well as for both histology subgroups).
Ilson et al. (2011)	Phase II	Total number: 30 patients ESCC: 13.	Erlotinib in patients with previously treated squamous cell and adenocarcinoma of the esophagus	Overall, erlotinib had limited activity in patients with esophageal cancer with some responses observed in ESCC.
Rodriguez et al. (2010)	Phase II	Total number: 173 ESCC: 19	Perioperative concurrent chemotherapy, gefitinib, and hyperfractionated radiation followed by maintenance gefitinib in locoregionally advanced esophagus and gastroesophageal junction cancer.	Gefitinib did not worsen CCRT toxicity; maintenance gefitinib proved difficult.
Zhai et al. (2013)	Phase II (pilot study)	18 patients	Concurrent erlotinib and radiotherapy for chemoradiotherapy-intolerant ESCC patients	For ESCC patients who cannot tolerate chemoradiotherapy, concurrent erlotinib and radiotherapy were tolerable and effective.
Huang et al. (2016)	Phase II	281 patients	Icotinib in Patients with Pretreated Advanced ESCC with EGFR overexpression or EGFR Gene Amplification	Overall, icotinib showed favorable activity in patients with advanced, previously treated ESCC with EGFR overexpression or amplification (in terms of response rate, overall survival and progression-free survival).
Janmmat et al. (2006)	Phase II	Total number: 36 patients ESCC: 9	Gefitinib in second-line treatment of advanced esophageal cancer patients	Overall, gefitinib has a modest activity in second-line treatment of advanced esophageal cancer. However, the patient outcome was significantly better in female patients and in patients demonstrating high EGFR expression or ESCC histology.
ii. VEGF/VEGFR-targeting agents:				
Janjigan et al. (2015)	Phase II	Total number: 35 patients ESCC: 5 patients	Sorafenib in chemotherapy-refractory esophageal carcinoma	For all patients: 8 week Kaplan-Meier estimated progression-free survival (PFS) was 61% (90% CI 45 to 73%). Median PFS was 3.6 months (95% CI 1.8 to 3.9 months), with median overall survival OS 9.7 months (95% CI 5.9 to 11.6 months).
Horgan et al. (2016)	Phase II	Total number: 61 patients ESCC: 12 patients	Adjuvant sunitinib following chemoradiotherapy and surgery for locally advanced esophageal cancer	For all patients: median survival was 26 months with a 2 and 3-year survival rate of 52% and 35%.

RTKs alone or in combination with other treatments (e.g. chemotherapy or radiotherapy) have achieved breakthrough advances in the management of a number of hard-to-treat malignancies (e.g. melanoma, renal cell carcinoma or oncogene driven non-small cell lung cancer) [15, 16].

#### Epidermal growth factor receptor (EGFR) in ESCC

The epidermal growth factor receptor (EGFR or ERBB1) is a surface protein, a member of the ERBB growth factor receptor family, which initiates signal transduction by activation of a receptor-associated tyrosine kinase (TK); ERBB family also includes ERBB2 (Her2), ERBB3, and ERBB4. The members of the EGFR family have three regions, a transmembrane domain, an extracellular ligand binding region, and an intracellular region with TK activity [17]. These members have been reported to play an important role in tumor cell proliferation, migration, angiogenesis and progression towards metastasis. Therefore, it has become an important therapeutic target in NSCLC, breast cancer and HNSCC.

EGFR overexpression and amplification was frequently observed in ESCC and correlated with advanced tumor stage and poor prognosis [18]. Among some ESCC cases, not only EGFR but EGF ligand was also expressed proving the involvement of an autocrine loop [19–21].

Moreover, overexpression of HER2–4 has been reported to be present in 30–80% of the ESCC cases. Overexpression of EGFR was correlated with poor prognosis [22]. Overexpression of EGFR and its ligand EGF (epidermal growth factor) have been reported long back in ESCC cell lines (TE1, TE2 & TE8) [23]. Since then a lot of progress has been made in the field. The abundance of the EGFR was reported to be 20-fold higher in esophageal cancer as compared with normal esophageal mucosa [24].

Overexpression of EGFR was observed specifically in 68% of the ESCC patients and it was significantly correlated with clinical stage, tumor invasion, and poor survival outcome [25]. In a radioimmunoassay, a positive correlation was found between overexpression of EGFR and poor prognosis in primary ESCC tumors [26]. EGFR overexpression was further associated with lymph node metastasis as well [27].

The *EGFR* amplification has been associated with diseases outcome in ESCC. ESCC patients with low copy number observed to have longer survival as compared with patients with high copy number of *EGFR* gene. *EGFR* amplification has been associated with advanced pathological stage and tumor lymph node metastasis [28].

Downstream pathways activated by signaling through EGFR family members include the MAP kinase pathway and the phosphatidylinositol 3-kinase (PI3K)/Akt pathway. The known ligands of the EGFR are EGF & transforming growth factor-alpha (TGF-α). Binding of a ligand to the EGFR causes it to dimerize either with itself or with another member of the ERBB family. Dimerization further leads to activation of TK, the downstream phosphorylation and activation of other effector signals [29]. In case of lung adenocarcinoma patients, somatic mutations present in the TK domain of *EGFR* responds well to TKIs targeting EGFR, but these TKI sensitive mutations in EGFR are very rare in ESCC patients [30, 31].

##### Small molecules or antibodies against EGFR used for ESCC

The expression of EGFR in ESCC varies between 33.3–72.1% with a significant association with occurrence of metastasis, involvement of lymph node and survival [32, 33–35]. The immnohistochemical study revealed that EGFR straining was restricted to the plasma membrane of the malignant cells in 71.2% of ESCC cases. In contrast, for pEGFR immunoreactivity was nuclear [36].

TKIs are a class of oral, minuscule molecules that inhibit ATP binding within the TK domain, leading to consummate inhibition of EGFR autophosphorylation and signal transduction. A number of anti-EGFR antibodies or small molecules have been tested in different malignancies including ESCC. In this context, there is a strong rationale for investigation of biological agents targeting EGFR family in ESCC. Gefitinib and erlotinib are EGFR-TKIs, which selectively block EGFR signaling through competitive reversible binding at intracellular EGFR-TK domain. EGFR has been studied extensively in relation to lung adenocarcinoma to target the mutant EGFR using erlotinib [37, 38]. TKIs of EGFR could be either reversible or irreversible. Among reversible TKIs are erlotinib, and gefitinib and the irreversible category includes afatinib, dacomitinib, and osimertinib.

##### Erlotinib and ESCC

The trade name for Erlotinib is Tarceva. Erlotinib has been used to treat the NSCLC patients [37]. Erlotinib bind to the TK domain of EGFR in a reversible manner and blocks the EGFR pathways by competing with ATP of the EGFR-TK domain. There were two clinical trials where Erlotinib was used for treatment of ESCC patients [39, 40]. The results of those trials have been summarized in Table 2.

##### Gefitinib and ESCC

Gefitinib (trade name Iressa) have been used for different types of solid malignancies. It inhibits EGFR *via* interruption of the EGFR signaling in the target cells. It is a reversible TKI of EGFR. Gefitinib was tested in ESCC TE8 cell line (with moderate type of tumor differentiation) [41]. In an *in vitro* study on ESCC cell lines (TE8, T.T and T.Tn), Gefitinib inhibited cellular proliferation in a dose-dependent manner, induced cell cycle arrest, inhibited ligand induced autophosphorylation of EGFR, downstream signaling pathways including Ras/Raf/MAPK and PI3K/Akt, and cell death [42].

Dutton and coworkers conducted a phase III study to evaluate gefitinib as a treatment for advanced esophageal cancer progressing on chemotherapy. Unfortunately, gefitinib was ineffective in proving overall survival (for both ESCC and EAD) [43]. There were two additional trials where gefitinib has been used for treatment of ESCC patients. The results of those have been summarized in Table 2 [43–45].

Gefitinib and erlotinib are reversible TKIs. These are reversible ATP mimetics that compete for ATP binding in the EGFR TK domain and competitively inhibit the binding of ATP to the EGFR TK domain [46]. This results in inhibition of EGFR phosphorylation (a post-translational modification) and downstream signaling. The irreversible EGFR TKIs are similarly ATP-mimetics, but those have the ability to bind covalently to cysteine residue at 797 position of EGFR [47].

##### Icotinib and ESCC

Icotinib is a small-molecule EGFR TKI, which binds to the ATP binding pocket of EGFR protein, and interrupts downstream signaling in a reversible manner [48]. EGFR overexpression and response to icotinib was studied in ESCC. An overexpression of EGFR was observed in 49% of the cases and it was correlated with clinical stage and lymph node metastasis significantly. Among a total of 62 ESCC patients treated with Icotinib, 17.6% were with high EGFR expression as compared with 0% patients with low to moderate expression of EGFR. Overall, the study suggests that overexpression of EGFR could be used in predicting the efficacy in icotinib treated ESCC patients [49].

Icotinib was clinically evaluated for the treatment of previously treated advanced ESCC patients who either had overexpression or amplification of EGFR in a single arm, multi-centric phase-II clinical trial [50]. The results have been summarized in Table 2.

##### Afatinib and ESCC

Afatinib (trade name Gilotrif in US) is a dual tyrosine kinase inhibitor of EGFR & ERRB2. These have been extensively used in lung adenocarcinoma, [38] lung squamous cell lung cancer, [51] and HNSCC [52]. In the xenograft derived from ESCC cell line KYSE270, treatment with Afatinib lead to reduction in the tumor volume in a dose dependent manner [53]. Afatinib was also tested in a pre-clinical study on ESCC cell lines (HKESC-2 and EC-1) where the IC50 was observed in lower μM range. The cell death indcued by Afatinib in ESCC cell lines was mediated by PARP-1 cleavage by the suicide proteases. There was no synergy observed between afatinib and the corner stone drug 5-flurouracil (5-FU) and cisplatin [53]. From a clinical viewpoint, afatinib was not yet formally evaluated in ESCC, hence its use cannot be justified in this indication.

##### Lapatinib and ESCC

Another TKI, Lapatinib is a reversible dual tyrosine kinase inhibitor of the EGFR and HER2 [54]. Lapatinib was tested in a panel of ESCC lines where it inhibited the Her2 phosphorylation; it’s amplification in HER2 overexpressing cells. Furthermore, Lapatinib inhibited proliferation of ESCC cells, induced cell death, and led to accumulation of EGFR and HER2 on the cell surface. In a combination of either trastuzumab or cetuximab with lapatinib, an increase in antibody-dependent cell-mediated cytotoxicity (ADCC) of 15–25%, and 15–30% was observed, respectively [55]. From a clinical standpoint, lapatinib was formally evaluated for esophageal/gastroesophageal/gastric adenocarcinoma with HER2 overexpression, but not in ESCC [56].

#### VEGFR (vascular endothelial growth factor receptor) in ESCC

Therapies directed against VEGF receptor (VEGFR) are the focus of major ongoing research in solid tumor malignancies. Folkman and others have provided compelling evidence linking tumor growth and metastasis with angiogenesis [57]. The ligand for VEGFRs is VEGF. There are three subtypes of VEGFR including VEGFR1, VEGFR2, and VEGFR3. These are alternative splice variants of VEGFR, an outcome of alternative RNA splicing [58]. Among identified angiogenic factors, VEGF is the most potent and specific and has been identified as a crucial regulator for both normal and pathologic angiogenesis. VEGF produces a number of biologic effects, including endothelial cell mitogenesis, migration and induction of proteinases, leading to remodeling of the extracellular matrix, increased vascular permeability, and maintenance of survival for the newly formed blood vessels [59]. VEGF exerts its angiogenic effects by binding to several high-affinity transmembrane receptors, most notably VEGFR1 and VEGFR2. An overexpression of VEGFR1 and VEGFR2 has been reported in ESCC cell lines. Furthermore, treatment of these cell lines with anti-VEGFR1/2 antibodies inhibits proliferation of ESCC cells denotes validity of VEGFRs as the genuine targets in ESCC [60]. Additionally, varied expression of *VEGFR1*, *VEGFR2*, and *VEGFR3* at transcriptional level was observed in ESCC [61]. VEGFR1 & VEGFR2 were reported in > 42% & 40% of the ESCC cases, respectively with cytoplasmic expression. VEGFR1-expressing cases were found to be associated with poor nodal status. There was no association between clinicopathological factors & prognosis with VEGFR2 expression [62]. VEGFR3 expression was significantly higher in sera of ESCC patients as compared with healthy donors [63].

In esophageal cancer, VEGF was overexpressed in 30–60% of patients, with several studies demonstrating a correlation between high levels of VEGF expression, advanced stage, and poor survival in patients undergoing esophagectomy. VEGF expression level is a predictor of tumor differentiation, TNM stage, distant metastasis, and overall survival (OS) in resectable ESCC cases [64]. In ESCC, expression of VEGF was associated with angiogenesis and progression of the disease [65, 66]. VEGF-targeting agents were evaluated mainly in EAD. Examples of small molecule VEGF-TKIs evaluated in clinical studies with mixed histology populations include sorafenib and sunitinib.

##### Sorafenib

One phase II study for sorafenib in chemotherapy-refractory esophageal carcinoma was carried out which incorporated both ESCC and EAD. Results for all patients suggested the ability of sorafenib to stabilize chemotherapy-refractory disease; however, these results were not stratified according to histology. The results of this clinical trial have been summarized in Table 2 [67].

##### Sunitinib

Sunitinib targets VEGFRs, PDGFR-β, and c-Kit [68]. A phase II clinical study evaluated adjuvant sunitinib following chemoradiotherapy for locally advanced esophageal cancer (both histologies). Results for all patients suggested that adjuvant sunitinib was poorly tolerated, with no signal of additional benefit over standard therapy. The results of the clinical trial has been summarized in Table-2 [69].

#### C-MET in ESCC

The MET proto-oncogene encode for protein c-MET, which belongs to RTK family. c-MET is also called as hepatocyte growth factor receptor (HGFR). It gets activated upon binding to its ligand hepatocyte growth factor (HGF). MET has been reported to overexpress in ESCC [70]. MET expression was observed in ~ 21% of the ESCC cases and interestingly it was correlated with PD-L1 (a ligand for PD1 receptor) expression [71].

MET is an emerging target and TK receptor for HGF [72]. MET had been reported in ≥ 50% of the ESCC cases at mRNA and protein levels [73]. Amplification of *MET* oncogene was found in 4–10% of gastric cancer cases [74]. Activation of the MET oncogene leads to multiple downstream pathways that promote the cancer phenotype. The most common c-MET small molecule inhibitor evaluated in gastrointestinal cancers is tivantinib. The principal clinical experiences with c-MET inhibitors were with gastric/gastroesophageal/esophageal adenocarcinomas [75, 76]. On the other hand, there are no fully published clinical data about c-MET TKIs in ESCC till now.

#### AXL in ESCC

AXL is a receptor tyrosine kinase, which belongs to TYRO3/AXL/MER. Overexpression of AXL has been reported in large number of malignancies including lung, colorectal [77], liver, oral squamous cell carcinoma (OSCC) [54], cutaneous squamous cell carcinoma [78], breast [79], HNSCC [80], pancreatic [81], and EAD [82]. In ESCC, expression of AXL kinase was observed in 80% of the total ESCC cases and correlated with the progression of the diseases [83]. In an in vitro model of ESCC, cells were found to be preferentially sensitive to foretinib (a c-MET, AXL and vascular endothelial growth factor receptor inhibitor) than lapatinib (HER2 inhibitor). Interestingly, both the agents had synergistic effect together indicates a possibility to use them together in vivo for an effective option in ESCC patients [83]. In ESCC cell lines (KYSE70 and KYSE180), AXL was playing an important as it exerted resistance towards PI3Kα *via* EGFR/PKC/mTOR pathway [84]. From a clinical perspective, none of the AXL inhibitors reached final phases of drug development in the indication of ESCC.

#### ALK in ESCC

Anaplastic Lymphoma Kinase (ALK) is also known as ALK tyrosine kinase receptor or CD246. Gene fusion may lead to exchange between two genes of either genetic code or regulatory DNA sequences. The translated products of gene fusion have been proved to be very important in cancer research [85]. A protein can be product of gene fusion, which could give rise to it by joining parts of two different genes. Some of the classical gene fusion examples involving a kinase-coding gene are *EML4-ALK* in lung adenocarcinoma [86], *ALK-RET* in colorectal cancers [87], and *VCL-ALK* in renal cell carcinoma (RCC) [88]. Similar cases were observed in ESCC, where fusion protein TPM4-ALK was detected in two separate proteomics based studies [89–91]. There is a need to study these gene fusion events associated with ALK to define their exact function and significance in relation to ESCC in different populations. However, beyond the basic science findings, ALK inhibitors were not formally evaluated in the setting of ESCC and no recommendation can be made about the use of any of these agents.

#### Protein tyrosine kinase 7 in ESCC

Protein tyrosine kinase 7 (PTK7) is an orphan TK, it belongs to the category of pseudokinases as some of the key residues essential for catalytic activity of PTK7 are missing in its kinase domain [92]. PTK7 also known as colon carcinoma kinase-4 (CCK-4) [93]. Overexpression of PTK7 has been reported in a number of different malignancies including oral tongue squamous cell carcinoma (OTSCC) [94], colorectal [95], and intrahepatic cholangiocarcinoma [96]. PTK7 overexpression has been reported in ~ 60% of the total ESCC cases. Its overexpression was correlated with poor prognosis of ESCC [97]. PTK7 increases invasive behavior of ESCC cells *via* NF-κB signaling when it is in catalytically defective form [98]. Furthermore, ESCC cell lines with higher expression of PTK7 have comparatively more refractive behaviors to radiation as compared with ESCC cells with low levels of PTK7 as evident with induction of apoptosis upon PTK7 knockdown in irradiated ESCC cells. The resistance to radiation in ESCC cells was regulated by PTK7 through NF-κB (nuclear factor-kappa B) [99]. PTK7 can act as a co-receptor with other RTKs like VEGFR1 to regulate other signaling pathways [100]. Till now, none of the agents targeting PTK7 were approved for the management of ESCC.

#### Insulin-like growth Factor-1 receptor in ESCC

The insulin-like growth factor type 1 receptor (IGF-1R) is a member of the receptor tyrosine kinases (RTK) family [101]. IGFIR is a tyrosine kinase that was significantly higher in adenomatous polyps and carcinoma as compared with healthy controls, and a positive correlation was observed between serum IGF1 and mucosal *IGF1R* mRNA expression in the polyps [102].

An improved sensitivity to radiation was found upon silencing IGF1R at in vitro and in vivo levels in ESCC cell lines [103]. Figitumumab (CP-751871, CP), an anti-IGF1R antibody (a human IgG2 monoclonal antibody, MAB) was screened in ESCC cell lines [104]. IGF1R and its ligands were found as overexpressed in ESCC as compared with normal epithelia [105, 106]. IGF1 contributes to resistant to chemotherapy agents used currently in clinic in ESCC and other cancers. Hence, there is a need of further investigations to estimate the exact role of IGF1R-IGF axis in ESCC [104]. There are  established clinical development programs for IGF1R inhibitors among patients with gastric or gastro-intestinal adenocarcinoma [107], but little has been done (from the clinical perspective) in the indication of ESCC.

#### Platelet-derived growth factor receptor in ESCC

The platelet-derived growth factor receptor (PDGFR) is another member of RTK family [101]. PDGFR subtypes are PDGFRα, and PDGFRß. Varied expressions of PDGFRα, and PDGFRß have been reported in ESCC cells [61]. Expression of PDGFRα was studied in cancer-associated fibroblast derived from ESCC patients and observed as an essential factor in ESCC progression; and expression of PDGFRβ was found to be associated with poorly differentiated tumors but not with prognosis [108]. Additionally, a downstream regulator of PDGFR stability small glutamine-rich tetratricopeptide repeat-containing protein alpha (SGTA) was upregulated in ESCC as compared with adjacent normal epithelia. An overexpression of SGTA was correlated with tumor grade. Additionally, an association between expression of SGTA and Ki-67 (a proliferation marker) was found suggesting role of SGTA in the proliferation of ESCC cells [109].

## Perspective and future directions

Receptor tyrosine kinases play a very crucial role in the maintenance, growth and differentiation of cancer cells including EAD and ESCC. ESCC is a multifactorial disease, which remains a public health problem worldwide. Over the past decade, the treatment of ESCC has been evolving rapidly. In the past the performance of the systematic therapies in ESCC was disappointing. TKIs such as Erlotinib, and Afatinib led to a great success in treating lung adenocarcinoma patients. This gives a hope specially when a fair number of TKIs are in different phases of clinical trials and some are in pipeline for development.

Among many other challenges, malignancies treated with chemotherapy and/or radiotherapy develops resistance to these treatments and become more aggressive and tends to have recurrence of the disease. Furthermore, blocking RTKs with one antibody or small molecule could trigger malignant cells to choose the alternative route for signaling and eventually that could lead to survival. This suggests the need for being vigilant of other signaling pathways, which could get activated as an alternative route after TKIs treatment.

Keeping these points in mind, more studies preferably involving an integration of multidimensional aspect of high-throughput genomics, transcriptomics, and proteomics profiling with biomarker-matched targeted therapy either alone or in combination with immunotherapy are required to overcome this deadly disease and for improvement both in the prognosis and survival of the ESCC patients. A thorough investigation is required to explore the co-expression of RTKs in ESCC as this phenomenon is dependent on the ligand binding. A well-designed study could lead to information about what combination of TKIs could be used for these kinds of tumors. Additionally, a clear and better understanding of the tumor pathophysiology, biology of ESCC and mechanism of action of minuscule molecules or anti-tyrosine kinase receptor antibodies is required.

Further, three-dimensional (3D) model of ESCC where co-culture combination of primary ESCC cells with microenvironment component could reveal which signaling pathway or active kinase is driving the signaling of the ESCC-microenvironment milieu as that could be used as a potential therapeutic target for treatment options in ESCC. Last but not least, there is a clear need for phospho-tyrosine targeted proteomics studies in settings like iTRAQ, where primary ESCC samples could be used for identification of RTKs specific peptide/proteins in ESCC.

## Conclusions

RTKs have been investigated extensively for research in relation to gastro-intestinal malignancies and a number of TKIs including reversible (erlotinib, and gefitinib), and irreversible (afatinib, dacomitinib, and osimertinib) have been tested for their efficacy in different malignancies including ESCC. The cancerous cells also evolved as those learned how to this mechanism and overcome the barriers imposed by exogenous/ intrinsic perturbations. Additionally, there is a need for development of an in vitro and/or in vivo model to test the effect of nexus between tumor-microenvironment more specifically ESCC-microenvironment interaction, and its impact on RTK signaling. The tumor-microenvironment has been reported to be associated with TKIs resistance by providing pro-survival factors secreted by the cellular components of the microenvironment. Hence, it will be of significance to identify RTKs actively involved in tumor-microenvironment nexus or cell-tissue interactions in an in vitro and/or in vivo model. The success/failure of identifying these molecules involved in this biological nexus is directly dependent on the availability of new/novel technologies. There is an optimism in identifying the missing links/points of RTK signaling in ESCC as tools and techniques involved in ‘Omic’ technologies (genomics, transcriptomics, proteomics and metabolomics) could help us understanding answering these questions related with different biological dimensions of ESCC tumorigenesis if it gets integrated with system based approaches.

## Abbreviations

5-FU: 5-Flurouracil
ATP: Adenosine triphosphate
AXL: AXL receptor tyrosine kinase
BE: Barrett’s esophagus
CCK4: Colon carcinoma kinase-4
CLL: Chronic lymphocytic leukemia
CML: Chronic myeloid leukemia
EAD: Esophageal adenocarcinoma
EGF: Epidermal growth factor
EGFR: Epidermal growth factor receptor
ESCC: Esophageal squamous cell carcinoma
GER: Gastroesophageal reflux
HGF: Hepatocyte growth factor
HGFR: Hepatocyte growth factor receptor
HNSCC: Head and neck squamous cell carcinoma
IGF: Insulin growth factor
IGF1R: Insulin-like growth factor I receptor
iTRAQ: Isobaric tags for relative and absolute quantitation
MAB: Monoclonal antibody
MET: Tyrosine-protein kinase Met
NRTKs: Non-receptor tyrosine kinases
NSCLC: Non-small cell lung cancer
OS: Overall survival
OSCC: Oral squamous cell carcinoma
OTSCC: Oral tongue squamous cell carcinoma
PDGF: Platelet-derived growth factor
PDGFR: Platelet-derived growth factor receptor
PDL1: A ligand for PD1 receptor
PI3K: Phosphatidylinositol 3-kinase
PTKs: Protein tyrosine kinases
pY: phospho-tyrosine
RTKs: Receptor tyrosine kinases
SGTA: Small glutamine-rich tetratricopeptide repeat-containing protein alpha
SILAC: Stable isotope labeling with amino acids in cell culture
TGF-α: Transforming growth factor-alpha
TK: Tyrosine kinase
TKIs: Tyrosine kinase inhibitors
VEGF: Vascular endothelial growth factor
VEGFR: Vascular endothelial growth factor receptor

## References

[1] Kashyap MK, Marimuthu A, Kishore CJ, Peri S, Keerthikumar S, Prasad TS, Mahmood R, Rao S, Ranganathan P, Sanjeeviah RC (2009). Genomewide mRNA profiling of esophageal squamous cell carcinoma for identification of cancer biomarkers. Cancer Biol Ther.

[2] Testa U, Castelli G, Pelosi E. Esophageal Cancer: Genomic and Molecular Characterization, Stem Cell Compartment and Clonal Evolution. Medicines (Basel). 2017;4:67. 10.3390/medicines4030067 PMC5622402 28930282

[3] Ajani JA, D'Amico TA, Almhanna K, Bentrem DJ, Besh S, Chao J, Das P, Denlinger C, Fanta P, Fuchs CS (2015). Esophageal and esophagogastric junction cancers, version 1.2015. J Natl Compr Cancer Netw.

[4] Hunter T (2000). Signaling--2000 and beyond. Cell.

[5] Hunter T (2007). The age of crosstalk: phosphorylation, ubiquitination, and beyond. Mol Cell.

[6] Madhusudan S, Ganesan TS (2004). Tyrosine kinase inhibitors in cancer therapy. Clin Biochem.

[7] Deng F, Zhou K, Li Q, Liu D, Li M, Wang H, Zhang W, Ma Y (2016). iTRAQ-based quantitative proteomic analysis of esophageal squamous cell carcinoma. Tumour Biol.

[8] Zhang J, Zhi C, Zhen F, Yuan X, Jiao C, Zhu H, Zhu H, Feng Y. iTRAQ-Based Quantitative Proteomic Analyses of High Grade Esophageal Squamous Intraepithelial Neoplasia. Proteomics Clin Appl. 2017;11:1600167. 10.1002/prca.201600167 28816019

[9] Wang X, Peng Y, Xie M, Gao Z, Yin L, Pu Y, Liu R. Identification of extracellular matrix protein 1 as a potential plasma biomarker of ESCC by proteomic analysis using iTRAQ and 2D-LC-MS/MS. Proteomics Clin Appl. 2017;11:1600163. 10.1002/prca.201600163 28493612

[10] Kashyap MK, Harsha HC, Renuse S, Pawar H, Sahasrabuddhe NA, Kim MS, Marimuthu A, Keerthikumar S, Muthusamy B, Kandasamy K (2010). SILAC-based quantitative proteomic approach to identify potential biomarkers from the esophageal squamous cell carcinoma secretome. Cancer Biol Ther.

[11] Pawar H, Kashyap MK, Sahasrabuddhe NA, Renuse S, Harsha HC, Kumar P, Sharma J, Kandasamy K, Marimuthu A, Nair B (2011). Quantitative tissue proteomics of esophageal squamous cell carcinoma for novel biomarker discovery. Cancer Biol Ther.

[12] Mir SA, Rajagopalan P, Jain AP, Khan AA, Datta KK, Mohan SV, Lateef SS, Sahasrabuddhe N, Somani BL, Keshava Prasad TS (2015). LC-MS-based serum metabolomic analysis reveals dysregulation of phosphatidylcholines in esophageal squamous cell carcinoma. J Proteome.

[13] Syed N, Barbhuiya MA, Pinto SM, Nirujogi RS, Renuse S, Datta KK, Khan AA, Srikumar K, Prasad TS, Kumar MV (2015). Phosphotyrosine profiling identifies ephrin receptor A2 as a potential therapeutic target in esophageal squamous-cell carcinoma. Proteomics.

[14] Ogawa R, Ohtsuka M, Sasadaira H, Hirasa M, Yabe H, Uchida H, Watanabe Y (1985). Increase of phosphotyrosine-containing proteins in human carcinomas. Jpn J Cancer Res.

[15] Ettinger DS, Wood DE, Aisner DL, Akerley W, Bauman J, Chirieac LR, D'Amico TA, DeCamp MM, Dilling TJ, Dobelbower M (2017). Non-small cell lung cancer, version 5.2017, NCCN clinical practice guidelines in oncology. J Natl Compr Cancer Netw.

[16] Coit DG, Thompson JA, Algazi A, Andtbacka R, Bichakjian CK, Carson WE, Daniels GA, DiMaio D, Fields RC, Fleming MD (2016). NCCN guidelines insights: melanoma, version 3.2016. J Natl Compr Cancer Netw.

[17] Roskoski R (2014). ErbB/HER protein-tyrosine kinases: structures and small molecule inhibitors. Pharmacol Res.

[18] Kim HS, Kim SM, Kim H, Pyo KH, Sun JM, Ahn MJ, Park K, Keam B, Kwon NJ, Yun HJ (2015). Phase II clinical and exploratory biomarker study of dacomitinib in recurrent and/or metastatic esophageal squamous cell carcinoma. Oncotarget.

[19] Hunts J, Ueda M, Ozawa S, Abe O, Pastan I, Shimizu N (1985). Hyperproduction and gene amplification of the epidermal growth factor receptor in squamous cell carcinomas. Jpn J Cancer Res.

[20] Ozawa S, Ueda M, Ando N, Abe O, Shimizu N (1987). High incidence of EGF receptor hyperproduction in esophageal squamous-cell carcinomas. Int J Cancer.

[21] Mukaida H, Toi M, Hirai T, Yamashita Y, Toge T (1991). Clinical significance of the expression of epidermal growth factor and its receptor in esophageal cancer. Cancer.

[22] Lin G, Sun XJ, Han QB, Wang Z, Xu YP, Gu JL, Wu W, Zhang GU, Hu JL, Sun WY, Mao WM (2015). Epidermal growth factor receptor protein overexpression and gene amplification are associated with aggressive biological behaviors of esophageal squamous cell carcinoma. Oncol Lett.

[23] Yoshida K, Kyo E, Tsuda T, Tsujino T, Ito M, Niimoto M, Tahara E (1990). EGF and TGF-alpha, the ligands of hyperproduced EGFR in human esophageal carcinoma cells, act as autocrine growth factors. Int J Cancer.

[24] Mukaida H, Yamamoto T, Hirai T, Toi M, Nakamura T, Wada T, Yamashita Y, Kawano K, Niimoto M (1990). Expression of human epidermal growth factor and its receptor in esophageal cancer. Jpn J Surg.

[25] Song J, Shi W, Zhang Y, Sun M, Liang X, Zheng S (2016). Epidermal growth factor receptor and B7-H3 expression in esophageal squamous tissues correlate to patient prognosis. Onco Targets Ther.

[26] Kitagawa Y, Ueda M, Ando N, Ozawa S, Shimizu N, Kitajima M (1996). Further evidence for prognostic significance of epidermal growth factor receptor gene amplification in patients with esophageal squamous cell carcinoma. Clin Cancer Res.

[27] Jiang D, Li X, Wang H, Shi Y, Xu C, Lu S, Huang J, Xu Y, Zeng H, Su J (2015). The prognostic value of EGFR overexpression and amplification in esophageal squamous cell carcinoma. BMC Cancer.

[28] Guo K, Wang WP, Jiang T, Wang JZ, Chen Z, Li Y, Zhou YA, Li XF, Lu Q, Zhang LJ (2016). Assessment of epidermal growth factor receptor mutation/copy number and K-ras mutation in esophageal cancer. J Thorac Dis.

[29] Seshacharyulu P, Ponnusamy MP, Haridas D, Jain M, Ganti AK, Batra SK (2012). Targeting the EGFR signaling pathway in cancer therapy. Expert Opin Ther Targets.

[30] Lin DC, Hao JJ, Nagata Y, Xu L, Shang L, Meng X, Sato Y, Okuno Y, Varela AM, Ding LW (2014). Genomic and molecular characterization of esophageal squamous cell carcinoma. Nat Genet.

[31] Gao YB, Chen ZL, Li JG, Hu XD, Shi XJ, Sun ZM, Zhang F, Zhao ZR, Li ZT, Liu ZY (2014). Genetic landscape of esophageal squamous cell carcinoma. Nat Genet.

[32] Hanawa M, Suzuki S, Dobashi Y, Yamane T, Kono K, Enomoto N, Ooi A (2006). EGFR protein overexpression and gene amplification in squamous cell carcinomas of the esophagus. Int J Cancer.

[33] Itakura Y, Sasano H, Shiga C, Furukawa Y, Shiga K, Mori S, Nagura H (1994). Epidermal growth factor receptor overexpression in esophageal carcinoma. An immunohistochemical study correlated with clinicopathologic findings and DNA amplification. Cancer.

[34] Shimada Y, Imamura M, Watanabe G, Uchida S, Harada H, Makino T, Kano M (1999). Prognostic factors of oesophageal squamous cell carcinoma from the perspective of molecular biology. Br J Cancer.

[35] Gibault L, Metges JP, Conan-Charlet V, Lozac'h P, Robaszkiewicz M, Bessaguet C, Lagarde N, Volant A (2005). Diffuse EGFR staining is associated with reduced overall survival in locally advanced oesophageal squamous cell cancer. Br J Cancer.

[36] Hoshino M, Fukui H, Ono Y, Sekikawa A, Ichikawa K, Tomita S, Imai Y, Imura J, Hiraishi H, Fujimori T (2007). Nuclear expression of phosphorylated EGFR is associated with poor prognosis of patients with esophageal squamous cell carcinoma. Pathobiology.

[37] Guha U, Chaerkady R, Marimuthu A, Patterson AS, Kashyap MK, Harsha HC, Sato M, Bader JS, Lash AE, Minna JD (2008). Comparisons of tyrosine phosphorylated proteins in cells expressing lung cancer-specific alleles of EGFR and KRAS. Proc Natl Acad Sci U S A.

[38] Zhang X, Maity T, Kashyap MK, Bansal M, Venugopalan A, Singh S, Awasthi S, Marimuthu A, Charles Jacob HK, Belkina N (2017). Quantitative tyrosine Phosphoproteomics of epidermal growth factor receptor (EGFR) tyrosine kinase inhibitor-treated lung adenocarcinoma cells reveals potential novel biomarkers of therapeutic response. Mol Cell Proteomics.

[39] Ilson DH, Kelsen D, Shah M, Schwartz G, Levine DA, Boyd J, Capanu M, Miron B, Klimstra D (2011). A phase 2 trial of erlotinib in patients with previously treated squamous cell and adenocarcinoma of the esophagus. Cancer.

[40] Zhai Y, Hui Z, Wang J, Zou S, Liang J, Wang X, Lv J, Chen B, Zhu H, Wang L (2013). Concurrent erlotinib and radiotherapy for chemoradiotherapy-intolerant esophageal squamous cell carcinoma patients: results of a pilot study. Dis Esophagus.

[41] Teraishi F, Kagawa S, Watanabe T, Tango Y, Kawashima T, Umeoka T, Nisizaki M, Tanaka N, Fujiwara T (2005). ZD1839 (Gefitinib, 'Iressa'), an epidermal growth factor receptor-tyrosine kinase inhibitor, enhances the anti-cancer effects of TRAIL in human esophageal squamous cell carcinoma. FEBS Lett.

[42] Hara F, Aoe M, Doihara H, Taira N, Shien T, Takahashi H, Yoshitomi S, Tsukuda K, Toyooka S, Ohta T, Shimizu N (2005). Antitumor effect of gefitinib ('Iressa') on esophageal squamous cell carcinoma cell lines in vitro and in vivo. Cancer Lett.

[43] Dutton SJ, Ferry DR, Blazeby JM, Abbas H, Dahle-Smith A, Mansoor W, Thompson J, Harrison M, Chatterjee A, Falk S (2014). Gefitinib for oesophageal cancer progressing after chemotherapy (COG): a phase 3, multicentre, double-blind, placebo-controlled randomised trial. Lancet Oncol.

[44] Janmaat ML, Gallegos-Ruiz MI, Rodriguez JA, Meijer GA, Vervenne WL, Richel DJ, Van Groeningen C, Giaccone G (2006). Predictive factors for outcome in a phase II study of gefitinib in second-line treatment of advanced esophageal cancer patients. J Clin Oncol.

[45] Rodriguez CP, Adelstein DJ, Rice TW, Rybicki LA, Videtic GM, Saxton JP, Murthy SC, Mason DP, Ives DI (2010). A phase II study of perioperative concurrent chemotherapy, gefitinib, and hyperfractionated radiation followed by maintenance gefitinib in locoregionally advanced esophagus and gastroesophageal junction cancer. J Thorac Oncol.

[46] Engelman JA, Janne PA (2008). Mechanisms of acquired resistance to epidermal growth factor receptor tyrosine kinase inhibitors in non-small cell lung cancer. Clin Cancer Res.

[47] Jia Y, Yun CH, Park E, Ercan D, Manuia M, Juarez J, Xu C, Rhee K, Chen T, Zhang H (2016). Overcoming EGFR(T790M) and EGFR(C797S) resistance with mutant-selective allosteric inhibitors. Nature.

[48] Sordella R, Bell DW, Haber DA, Settleman J (2004). Gefitinib-sensitizing EGFR mutations in lung cancer activate anti-apoptotic pathways. Science.

[49] Wang X, Niu H, Fan Q, Lu P, Ma C, Liu W, Liu Y, Li W, Hu S, Ling Y (2016). Predictive value of EGFR overexpression and gene amplification on icotinib efficacy in patients with advanced esophageal squamous cell carcinoma. Oncotarget.

[50] Huang J, Fan Q, Lu P, Ying J, Ma C, Liu W, Liu Y, Tan F, Sun Y (2016). Icotinib in patients with pretreated advanced esophageal squamous cell carcinoma with EGFR overexpression or EGFR gene amplification: a single-arm, multicenter phase 2 study. J Thorac Oncol.

[51] Hirsh V (2017). New developments in the treatment of advanced squamous cell lung cancer: focus on afatinib. Onco Targets Ther.

[52] Cohen EEW, Licitra LF, Burtness B, Fayette J, Gauler T, Clement PM, Grau JJ, Del Campo JM, Mailliez A, Haddad RI (2017). Biomarkers predict enhanced clinical outcomes with afatinib versus methotrexate in patients with second-line recurrent and/or metastatic head and neck cancer. Ann Oncol.

[53] Wong CH, Ma BB, Hui CW, Tao Q, Chan AT (2015). Preclinical evaluation of afatinib (BIBW2992) in esophageal squamous cell carcinoma (ESCC). Am J Cancer Res.

[54] Chiu KC, Lee CH, Liu SY, Chou YT, Huang RY, Huang SM, Shieh YS (2015). Polarization of tumor-associated macrophages and Gas6/Axl signaling in oral squamous cell carcinoma. Oral Oncol.

[55] Mimura K, Kono K, Maruyama T, Watanabe M, Izawa S, Shiba S, Mizukami Y, Kawaguchi Y, Inoue M, Kono T (2011). Lapatinib inhibits receptor phosphorylation and cell growth and enhances antibody-dependent cellular cytotoxicity of EGFR- and HER2-overexpressing esophageal cancer cell lines. Int J Cancer.

[56] Hecht JR, Bang YJ, Qin SK, Chung HC, Xu JM, Park JO, Jeziorski K, Shparyk Y, Hoff PM, Sobrero A (2016). Lapatinib in combination with Capecitabine plus Oxaliplatin in human epidermal growth factor receptor 2-positive advanced or metastatic gastric, esophageal, or gastroesophageal adenocarcinoma: TRIO-013/LOGiC--A randomized phase III trial. J Clin Oncol.

[57] Folkman J (1995). Angiogenesis in cancer, vascular, rheumatoid and other disease. Nat Med.

[58] Jin P, Zhang J, Sumariwalla PF, Ni I, Jorgensen B, Crawford D, Phillips S, Feldmann M, Shepard HM, Paleolog EM (2008). Novel splice variants derived from the receptor tyrosine kinase superfamily are potential therapeutics for rheumatoid arthritis. Arthritis Res Ther.

[59] Ferrara N (2009). Vascular endothelial growth factor. Arterioscler Thromb Vasc Biol.

[60] Xu WW, Li B, Lam AK, Tsao SW, Law SY, Chan KW, Yuan QJ, Cheung AL (2015). Targeting VEGFR1- and VEGFR2-expressing non-tumor cells is essential for esophageal cancer therapy. Oncotarget.

[61] Gockel I, Moehler M, Frerichs K, Drescher D, Trinh TT, Duenschede F, Borschitz T, Schimanski K, Biesterfeld S, Herzer K (2008). Co-expression of receptor tyrosine kinases in esophageal adenocarcinoma and squamous cell cancer. Oncol Rep.

[62] Kato H, Yoshikawa M, Miyazaki T, Nakajima M, Fukai Y, Masuda N, Fukuchi M, Manda R, Tsukada K, Kuwano H (2002). Expression of vascular endothelial growth factor (VEGF) and its receptors (Flt-1 and Flk-1) in esophageal squamous cell carcinoma. Anticancer Res.

[63] Su Mer A, Demir A, Kemik AS, Yavuz A, Suvak B, Du Lger AC, Purisa S, Kemik O (2017). Serum vascular endothelial growth factor receptor-3 levels in patients with esophageal squamous cell cancer. Ann Ital Chir.

[64] Hou X, Wei JC, Fu JH, Wang X, Luo RZ, He JH, Zhang LJ, Lin P, Yang HX (2015). Vascular endothelial growth factor is a useful predictor of postoperative distant metastasis and survival prognosis in esophageal squamous cell carcinoma. Ann Surg Oncol.

[65] Inoue K, Ozeki Y, Suganuma T, Sugiura Y, Tanaka S (1997). Vascular endothelial growth factor expression in primary esophageal squamous cell carcinoma. Association with angiogenesis and tumor progression. Cancer.

[66] Kleespies A, Guba M, Jauch KW, Bruns CJ (2004). Vascular endothelial growth factor in esophageal cancer. J Surg Oncol.

[67] Janjigian YY, Vakiani E, Ku GY, Herrera JM, Tang LH, Bouvier N, Viale A, Socci ND, Capanu M, Berger M, Ilson DH (2015). Phase II trial of Sorafenib in patients with chemotherapy refractory metastatic esophageal and gastroesophageal (GE) junction cancer. PLoS One.

[68] Morabito A, De Maio E, Di Maio M, Normanno N, Perrone F (2006). Tyrosine kinase inhibitors of vascular endothelial growth factor receptors in clinical trials: current status and future directions. Oncologist.

[69] Horgan AM, Darling G, Wong R, Guindi M, Liu G, Jonker DJ, Lister J, Xu W, MacKay HM, Dinniwell R (2016). Adjuvant sunitinib following chemoradiotherapy and surgery for locally advanced esophageal cancer: a phase II trial. Dis Esophagus.

[70] Xu Y, Peng Z, Li Z, Lu M, Gao J, Li Y, Li Y, Shen L (2015). Expression and clinical significance of c-met in advanced esophageal squamous cell carcinoma. BMC Cancer.

[71] Kim R, Keam B, Kwon D, Ock CY, Kim M, Kim TM, Kim HJ, Jeon YK, Park IK, Kang CH (2016). Programmed death ligand-1 expression and its prognostic role in esophageal squamous cell carcinoma. World J Gastroenterol.

[72] Smyth EC, Sclafani F, Cunningham D (2014). Emerging molecular targets in oncology: clinical potential of MET/hepatocyte growth-factor inhibitors. Onco Targets Ther.

[73] Janjigian YY, Tang LH, Coit DG, Kelsen DP, Francone TD, Weiser MR, Jhanwar SC, Shah MA (2011). MET expression and amplification in patients with localized gastric cancer. Cancer Epidemiol Biomark Prev.

[74] Marano L, Chiari R, Fabozzi A, De Vita F, Boccardi V, Roviello G, Petrioli R, Marrelli D, Roviello F, Patriti A (2015). C-met targeting in advanced gastric cancer: an open challenge. Cancer Lett.

[75] Abdel-Rahman O (2015). Targeting the hepatocyte growth factor/mesenchymal epithelial transition pathway in gastric cancer: biological rationale and clinical applications. Expert Rev Anticancer Ther.

[76] Pant S, Patel M, Kurkjian C, Hemphill B, Flores M, Thompson D, Bendell J (2017). A phase II study of the c-met inhibitor Tivantinib in combination with FOLFOX for the treatment of patients with previously untreated metastatic adenocarcinoma of the distal esophagus, gastroesophageal junction, or stomach. Cancer Investig.

[77] Uribe DJ, Mandell EK, Watson A, Martinez JD, Leighton JA, Ghosh S, Rothlin CV (2017). The receptor tyrosine kinase AXL promotes migration and invasion in colorectal cancer. PLoS One.

[78] Cichon MA, Szentpetery Z, Caley MP, Papadakis ES, Mackenzie IC, Brennan CH, O'Toole EA (2014). The receptor tyrosine kinase Axl regulates cell-cell adhesion and stemness in cutaneous squamous cell carcinoma. Oncogene.

[79] Gjerdrum C, Tiron C, Hoiby T, Stefansson I, Haugen H, Sandal T, Collett K, Li S, McCormack E, Gjertsen BT (2010). Axl is an essential epithelial-to-mesenchymal transition-induced regulator of breast cancer metastasis and patient survival. Proc Natl Acad Sci U S A.

[80] Brand TM, Iida M, Stein AP, Corrigan KL, Braverman CM, Coan JP, Pearson HE, Bahrar H, Fowler TL, Bednarz BP (2015). AXL is a logical molecular target in head and neck squamous cell carcinoma. Clin Cancer Res.

[81] Koorstra JB, Karikari CA, Feldmann G, Bisht S, Rojas PL, Offerhaus GJ, Alvarez H, Maitra A (2009). The Axl receptor tyrosine kinase confers an adverse prognostic influence in pancreatic cancer and represents a new therapeutic target. Cancer Biol Ther.

[82] Hector A, Montgomery EA, Karikari C, Canto M, Dunbar KB, Wang JS, Feldmann G, Hong SM, Haffner MC, Meeker AK (2010). The Axl receptor tyrosine kinase is an adverse prognostic factor and a therapeutic target in esophageal adenocarcinoma. Cancer Biol Ther.

[83] Hsieh MS, Yang PW, Wong LF, Lee JM (2016). The AXL receptor tyrosine kinase is associated with adverse prognosis and distant metastasis in esophageal squamous cell carcinoma. Oncotarget.

[84] Elkabets M, Pazarentzos E, Juric D, Sheng Q, Pelossof RA, Brook S, Benzaken AO, Rodon J, Morse N, Yan JJ (2015). AXL mediates resistance to PI3Kalpha inhibition by activating the EGFR/PKC/mTOR axis in head and neck and esophageal squamous cell carcinomas. Cancer Cell.

[85] Mertens F, Johansson B, Fioretos T, Mitelman F (2015). The emerging complexity of gene fusions in cancer. Nat Rev Cancer.

[86] Soda M, Choi YL, Enomoto M, Takada S, Yamashita Y, Ishikawa S, Fujiwara S, Watanabe H, Kurashina K, Hatanaka H (2007). Identification of the transforming EML4-ALK fusion gene in non-small-cell lung cancer. Nature.

[87] Lipson D, Capelletti M, Yelensky R, Otto G, Parker A, Jarosz M, Curran JA, Balasubramanian S, Bloom T, Brennan KW (2012). Identification of new ALK and RET gene fusions from colorectal and lung cancer biopsies. Nat Med.

[88] Debelenko LV, Raimondi SC, Daw N, Shivakumar BR, Huang D, Nelson M, Bridge JA (2011). Renal cell carcinoma with novel VCL-ALK fusion: new representative of ALK-associated tumor spectrum. Mod Pathol.

[89] Moghanibashi M, Jazii FR, Soheili ZS, Zare M, Karkhane A, Parivar K, Mohamadynejad P (2012). Proteomics of a new esophageal cancer cell line established from Persian patient. Gene.

[90] Du XL, Hu H, Lin DC, Xia SH, Shen XM, Zhang Y, Luo ML, Feng YB, Cai Y, Xu X (2007). Proteomic profiling of proteins dysregulted in Chinese esophageal squamous cell carcinoma. J Mol Med (Berl).

[91] Jazii FR, Najafi Z, Malekzadeh R, Conrads TP, Ziaee AA, Abnet C, Yazdznbod M, Karkhane AA, Salekdeh GH (2006). Identification of squamous cell carcinoma associated proteins by proteomics and loss of beta tropomyosin expression in esophageal cancer. World J Gastroenterol.

[92] Mossie K, Jallal B, Alves F, Sures I, Plowman GD, Ullrich A (1995). Colon carcinoma kinase-4 defines a new subclass of the receptor tyrosine kinase family. Oncogene.

[93] Keshava Prasad TS, Goel R, Kandasamy K, Keerthikumar S, Kumar S, Mathivanan S, Telikicherla D, Raju R, Shafreen B, Venugopal A (2009). Human protein reference database--2009 update. Nucleic Acids Res.

[94] Dong Y, Chen X, Li H, Ni Y, Han W, Wang J (2017). PTK7 is a molecular marker for metastasis, TNM stage, and prognosis in oral tongue squamous cell carcinoma. Pol J Pathol.

[95] Tian X, Yan L, Zhang D, Guan X, Dong B, Zhao M, Hao C (2016). PTK7 overexpression in colorectal tumors: Clinicopathological correlation and prognosis relevance. Oncol Rep.

[96] Jin J, Ryu HS, Lee KB, Jang JJ (2014). High expression of protein tyrosine kinase 7 significantly associates with invasiveness and poor prognosis in intrahepatic cholangiocarcinoma. PLoS One.

[97] Shin WS, Kwon J, Lee HW, Kang MC, Na HW, Lee ST, Park JH (2013). Oncogenic role of protein tyrosine kinase 7 in esophageal squamous cell carcinoma. Cancer Sci.

[98] Shin WS, Hong Y, Lee HW, Lee ST (2016). Catalytically defective receptor protein tyrosine kinase PTK7 enhances invasive phenotype by inducing MMP-9 through activation of AP-1 and NF-kappaB in esophageal squamous cell carcinoma cells. Oncotarget.

[99] Park M, Yoon HJ, Kang MC, Kwon J, Lee HW (2016). PTK7 regulates radioresistance through nuclear factor-kappa B in esophageal squamous cell carcinoma. Tumour Biol.

[100] Wagner G, Peradziryi H, Wehner P, Borchers A (2010). PlexinA1 interacts with PTK7 and is required for neural crest migration. Biochem Biophys Res Commun.

[101] Favelyukis S, Till JH, Hubbard SR, Miller WT (2001). Structure and autoregulation of the insulin-like growth factor 1 receptor kinase. Nat Struct Biol.

[102] Zhang R, Xu GL, Li Y, He LJ, Chen LM, Wang GB, Lin SY, Luo GY, Gao XY, Shan HB (2013). The role of insulin-like growth factor 1 and its receptor in the formation and development of colorectal carcinoma. J Int Med Res.

[103] Zhao H, Gu X (2014). Silencing of insulin-like growth factor-1 receptor enhances the radiation sensitivity of human esophageal squamous cell carcinoma in vitro and in vivo. World J Surg Oncol.

[104] Zhang T, Shen H, Dong W, Qu X, Liu Q, Du J (2014). Antitumor effects and molecular mechanisms of figitumumab, a humanized monoclonal antibody to IGF-1 receptor, in esophageal carcinoma. Sci Rep.

[105] Liu YC, Leu CM, Wong FH, Fong WS, Chen SC, Chang C, Hu CP (2002). Autocrine stimulation by insulin-like growth factor I is involved in the growth, tumorigenicity and chemoresistance of human esophageal carcinoma cells. J Biomed Sci.

[106] Imsumran A, Adachi Y, Yamamoto H, Li R, Wang Y, Min Y, Piao W, Nosho K, Arimura Y, Shinomura Y (2007). Insulin-like growth factor-I receptor as a marker for prognosis and a therapeutic target in human esophageal squamous cell carcinoma. Carcinogenesis.

[107] Abdel-Rahman O (2015). Insulin-like growth factor pathway aberrations and gastric cancer; evaluation of prognostic significance and assessment of therapeutic potentials. Med Oncol.

[108] Ha SY, Yeo SY, Xuan YH, Kim SH (2014). The prognostic significance of cancer-associated fibroblasts in esophageal squamous cell carcinoma. PLoS One.

[109] Yang X, Cheng L, Li M, Shi H, Ren H, Ding Z, Liu F, Wang Y, Cheng C (2014). High expression of SGTA in esophageal squamous cell carcinoma correlates with proliferation and poor prognosis. J Cell Biochem.

